# Safety and procedural success of daycase-based endovascular procedures in lower extremity arteries of patients with peripheral artery disease: a systematic review and meta-analysis

**DOI:** 10.1016/j.eclinm.2024.102788

**Published:** 2024-09-05

**Authors:** Lydia Hanna, Alexander D. Rodway, Puneet Garcha, Luci Maynard, Janane Sivayogi, Oliver Schlager, Juraj Madaric, Vinko Boc, Lucas Busch, Martin B. Whyte, Simon S. Skene, Jenny Harris, Christian Heiss

**Affiliations:** a Vascular Medicine Department, Surrey and Sussex Healthcare NHS Trust, Redhill, UK; b Department of Surgery and Cancer, Imperial College London, London, UK; c Department of Clinical and Experimental Medicine, Faculty of Health and Medical Sciences, University of Surrey, Guildford, UK; d Brighton and Sussex University Hospitals NHS Trust, Brighton, UK; e Division of Angiology, Department of Medicine II, Medical University of Vienna, Austria; f Department of Angiology, Comenius University and National Institute of Cardiovascular Diseases, Bratislava, Slovakia; g Division of Cardiology, Pulmonology and Vascular Medicine, Medical Faculty, Heinrich-Heine University, Düsseldorf, Germany; h Clinical Department of Vascular Diseases, University Medical Centre Ljubljana, Ljubljana, Slovenia; i School of Health Sciences, Faculty of Health and Medical Sciences, University of Surrey, Guildford, UK

**Keywords:** Daycase angioplasty, Chronic limb-threatening ischaemia, Peripheral artery disease, Endovascular, Technical success

## Abstract

**Background:**

Timely and economic provision of revascularisation procedures is a major healthcare need. We aimed to examine the safety and efficacy of daycase-based lower extremity endovascular revascularisation procedures in patients with peripheral artery disease.

**Methods:**

In this systematic review and meta-analysis, we searched MEDLINE and Embase for studies from Jan 01, 2000 through Apr 01, 2024 reporting complications of lower limb endovascular revascularisation procedures with same-day discharge. Eligibility-criteria, complications, and patient characteristics were extracted, methodological quality assessed (adapted Newcastle–Ottawa Scale), and meta-analyses of complications and technical success performed to provide pooled estimates. This study is registered with PROSPERO, CRD42022316466.

**Findings:**

Thirty observational studies (17 retrospective, 13 prospective) and 1 RCT reported 2427 minor and 653 major complications after 99,600 daycase procedures (93,344 patients). Eighteen studies reported daycase eligibility-criteria including ‘responsible adult companion’ (78%), ‘proximity to hospital’, and ‘telephone availability’ and excluding unstable and severe co-morbidities, offset coagulation, and severe chronic kidney disease. Pooled incidences of minor (4.7% [95% CI 3.8–5.6%], *I*^2^ = 96%) and major (0.64% [95% CI 0.48–0.79%], *I*^*2*^ = 46%) complications were low and technical success high (93% [95% CI 91–96%], *I*^2^ = 97%). Most complications were related to the puncture site. Pooled conversion-to-hospitalisation rates and re-admission after discharge were 1.6% (95% CI 1.1–2.2%, *I*^2^ = 82%) and 0.11% (95% CI 0.095–0.23%, *I*^2^ = 97%), respectively. Meta-regression identified that minor complications decreased since 2000. Male sex and coronary artery disease were associated with more frequent, and higher age and closure device use with less minor complications. Diabetes mellitus and chronic kidney disease were associated with less major complications. Six studies reported complication rates both in daycases and inpatients and there was no significant difference (−0.8% [95% CI −1.9 to 0.3%]).

**Interpretation:**

After careful evaluation of eligibility, lower limb angioplasty can be performed safely with high technical success in a daycase setting. Most complications arise from the puncture site and not the procedure itself highlighting the importance of optimal access site management. The heterogeneity between studies warrants standardised monitoring of complications and outcomes.

**Funding:**

10.13039/100019599European Partnership on Metrology, co-financed from European Union’s Horizon Europe Research and Innovation Programme and 10.13039/100014013UK Research and Innovation, and 10.13039/501100000265Medical Research Council.

**Research in context**

**Evidence before this study**

In a preliminary search of Pubmed, Embase and Medline, we scoped the existing evidence on the safety and efficacy of revascularisation procedures for treatment of lower limb peripheral artery disease (PAD) between January 2020 and March 2023 in English language literature. Our search terms included ‘angioplasty’ Or ‘revascularisation’ AND ‘daycase’ OR ‘same day discharge’ OR ‘outpatients’ AND ‘peripheral artery disease’ OR ‘Chronic limb-threatening isch∗emia’ OR ‘claudication’. We identified a number of observational studies reporting complications in patients with PAD treated as outpatients. One French guideline document published in 2019 included a systematic review including 12 studies but did not provide a structured review of the literature including eligibility criteria used, meta-analyse the results, report pooled estimates of complication rates and technical success, or compare with complications in inpatients.

**Added value of this study**

The systematic review summarises daycase eligibility criteria from 18 studies. The meta-analysis provides pooled estimates of reported technical success (93%), major (0.64%) and minor complication (4.7%) rates in 30 observational studies (17 retrospective, 13 prospective) and 1 RCT reported after 99,600 daycase procedures (93,344 patients). In addition, complications were assessed separately in retrospective and prospective studies and after re-adjudication to standardised criteria by CIRSE and SIR. A meta-regression analysis identified factors that are both positively and negatively associated with complications. In the six studies that reported data for both daycases and inpatients complications did not differ.

**Implications of all the available evidence**

The systematic review highlights the importance of social factors for selection of patients. Despite large heterogeneity between studies in particular for minor complications, only 24 studies being of fair to high quality and mostly observational studies, the meta-analysis demonstrates overall low major complication rates (0.7%) and high technical success (93%) across a wide range of patients including chronic limb-threatening ischaemia and variety of settings. Most complications arise from the (mainly femoral) puncture site and not the procedure itself highlighting the importance of optimal access site management. The heterogeneity between studies warrants monitoring of complications according to standard criteria and outcomes. These data can be used as part of the evidence base for clinical decision making, help inform patients during informed consent, and for guideline development.

## Introduction

Peripheral artery disease (PAD) represents a considerable health burden worldwide affecting as many as 1 in 4 adults.^1^^,^^2^ As the population ages and prevalence of risk factors in particular diabetes mellitus increase, we are likely to see a rise in both the prevalence and the incidence of PAD. PAD represents a spectrum ranging from intermittent limb claudication, through to rest pain and tissue loss termed chronic limb-threatening ischaemia (CLTI), often as a result of multi-level severe stenotic and/or occlusive atherosclerotic disease that leads to significant limitation of blood flow to the legs. In particular in CLTI, timely diagnosis and revascularisation are needed as the 1-year risk of mortality or major amputation is as high as 50%.^3^ In a large proportion of patients revascularisation can be performed endovascular^4^ and the numbers of endovascular procedures are increasing while amputations are decreasing.^5^ An ‘endovascular first (if technically feasible)’ approach is associated with improved amputation-free survival in patients with CLTI^6^ and is endorsed by vascular societies.^7^ The timely performance of revascularisation can be limited by increasing pressures on the healthcare system leading to long waiting times.

Daycase-based provision of angioplasties may be a solution to increase capacity to meet the need and optimise scarce healthcare resources. The feasibility and safety of daycase peripheral angioplasty is not a new concept but has in fact been reported since 1983. While today in many countries patients are still routinely admitted and treated as inpatients, in some countries angioplasties are performed routinely on a daycase basis. For instance in the UK, the delivery of endovascular therapy through day care is part of the *Best Practice Clinical Care Pathway*.^8^ In other countries, vascular societies have raised concerns regarding patient safety and summarised organisational and patient specific factors to be considered for daycase procedures.^9^ Given that PAD typically affects older, co-morbid patients who may be at high risk from major surgical revascularisation techniques, a first line (and potentially only available) treatment for this group of patients is with minimally invasive angioplasty and/or stent placement to restore arterial flow and prevent limb loss. However, there is no consensus on the eligibility of patients for same day discharge. The safety and effectiveness of same day discharge procedures has not been systematically evaluated as an evidence base for clinical decision making and guideline development.

Here, we aim to close this gap and provide a systematic review and meta-analysis to evaluate current literature in terms of identifying daycase eligibility criteria and provide pooled estimates of complications and technical success. We hypothesized that the complication rates are low and technical success is high.

## Methods

### Search strategy and selection criteria

The systematic review was undertaken in accordance with the *Preferred Reporting Items for Systematic Reviews and Meta-analyses* (PRISMA) guidelines.^10^ The methodology and prespecified protocol were registered on the international prospective register of systematic reviews (PROSPERO, registration CRD42022316466). Reporting follows the MOOSE reporting guidelines.

The initial literature search on MEDLINE and Embase was performed by authors (LH, PG, LM, JS) with help of an expert librarian using Ovid covering January 1st, 2000 to March 1st, 2023 and updated in April 1st, 2024. The search comprised a combination of the terms ‘angioplasty’ and ‘day-case’. Older studies were excluded as these studies are unlikely to reflect contemporary practice with the inclusion of new angioplasty techniques such as drug coated balloons. A hand search of reference lists in obtained studies was also undertaken to identify additional studies. Authors were not contacted. Only abstracts and unpublished studies were not included. The literature search strategy is presented in Supplementary Table S1.

Search results were imported into COVIDENCE for study selection and duplicate removal. Title and abstract screening were undertaken, data extracted and coded by four investigators (LH, PG, LM, JS). Any disagreements between reviewers were remedied through consensus discussion with the senior author (CH). The population of interest was patients with peripheral artery disease defined as intermittent limb claudication and/or CLTI. The intervention/exposure was day-case lower limb angioplasty, with/without inpatient comparator group. Outcomes included all complications as defined and reported by the respective study authors and technical and clinical success. Randomised and observational studies in the English language were eligible for inclusion and no translation tools were used to include non-English language literature articles. Studies with less than 5 participants were excluded to reduce risk of bias. Systematic reviews, review articles, case reports, editorials, and conference abstracts were excluded. Inclusion and exclusion criteria are provided in Supplementary Table S2.

### Data analysis

Data were extracted using a pre-defined standardised collection form that included publication information, study design, study demographics (age, sex, co-morbidities), procedural characteristics, complications, technical and clinical success. Author inclusion and exclusion criteria for ‘day-case’ and ‘complications’ (major/minor, if provided) were recorded. In case studies did not explicitly state minor and major complications separately, these were assigned based on best clinical judgement with consensus of the co-authors, aligning with the *Society of Interventional Radiology* (*SIR*) criteria. Complications were also organised according to the complication definitions by *Cardiovascular Interventional Radiology Society Europe* (*CIRSE*) and *SIR* (Supplementary Table S3).^11^^,^^12^

The methodological quality of each study was evaluated using an adapted version of the Newcastle Ottawa Scale (Supplementary Tables S4 and S5) across the following three domains: selection, comparability, and outcomes. Nine quality items were evaluated in each study, with a maximum total of 4 stars in selection, 2 stars in comparability, and 3 stars in outcome. A risk of bias table was synthesised grouping studies as poor (0 or 1 star in selection domain OR 0 stars in comparability domain OR 0 or 1 stars in outcome/exposure domain), fair (2 stars in selection domain AND 1 or 2 stars in comparability domain AND 2 or 3 stars in outcome/exposure domain), or good (3 or 4 stars in selection domain AND 1 or 2 stars in comparability domain AND 2 or 3 stars in outcome/exposure domain).

Statistical analyses were performed using the ‘meta-analysis’ function for continuous outcomes with pre-calculated effect sizes using SPSS 29 (IBM) to estimate pooled incidence of major and minor complications, technical success, and conversion to hospitalisation. A 95% confidence interval (CI) to synthesize meta-analysis forest plots for minor and major complications per study was employed. A generic inverse variance method with random effects model was utilised, to provide an estimate of the between-study variability accounting for potential heterogeneity, with the assumption that complication prevalence would be variable. Heterogeneity across the included studies was evaluated via the synthesised *I*^2^ statistic, with *I*^2^ between 50 and 75% considered as moderate heterogeneity and >75% as substantially heterogenous. Sensitivity analyses included a pooled prevalence of complications for studies quality rated as ‘good’, ‘fair’, and ‘poor’, as well as for the pooled incidence of major and minor complications, as defined by *CIRSE* and *SIR*. Random effects meta-regression was performed to evaluate which factors were associated with complications (independent variable) and could therefore help to explain heterogeneity. The factors investigated as dependent variables were year of publication, age of patients, sex (male vs female), proportions with diabetes mellitus and patients who were currently smoking, level of disease (percentage of IC vs CLTI patients), sheath used (>7 Fr) and proportion of intervention vs diagnostic procedure. Publication bias was assessed using Funnel plots and Egger’s test including parameters that reflect ‘baseline risk’ identified in meta-regression analysis (minor: publication year, age, male sex, CAD, closure device use, intervention; major: diabetes mellitus, CKD) as co-variates.

Finally, a meta-analysis comparing complications rates for studies reporting on complications of day-case patients and those who needed hospitalisation was also undertaken. Again, a random effects model with DerSimonian-Laird method was used to account for heterogeneity. The standard p-value of <0.05 was used to deem statistical significance of results. All analyses were performed with SPSS 29 (IBM).

### Role of the funding source

The funder of the study had no role in study design, data collection, data analysis, data interpretation, or writing of the report. All authors had full access to the data in the study and had final responsibility for the decision to submit for publication.

## Results

### Literature search

**Fig. 1:**
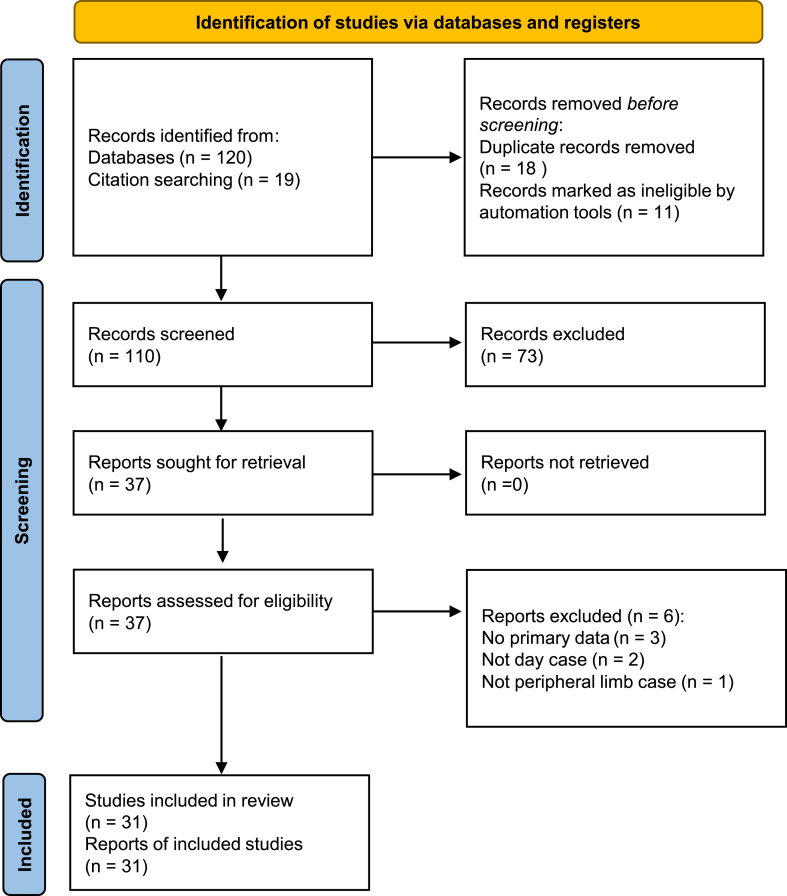
PRISMA flow chart to illustrate the process by which articles were selected or rejected for inclusion in the quantitative meta-analysis synthesis for this study.

The initial search identified 139 records. Following removal of duplicate records and adding records based on references found in the papers, 31 eligible studies were included in the quantitative meta-analysis.^13^, ^14^, ^15^, ^16^, ^17^, ^18^, ^19^, ^20^, ^21^, ^22^, ^23^, ^24^, ^25^, ^26^, ^27^, ^28^, ^29^, ^30^, ^31^, ^32^, ^33^, ^34^, ^35^, ^36^, ^37^, ^38^, ^39^, ^40^, ^41^, ^42^, ^43^ Fig. 1 shows the PRISMA Flow Diagram.

### Study characteristics

The 31 studies comprised 17 retrospective and 14 prospective,^17^^,^^18^^,^^22^^,^^23^^,^^25^, ^26^, ^27^^,^^29^^,^^37^^,^^38^^,^^42^ including 1 RCT^13^ with 99,600 daycase lower limb/peripheral angioplasty procedures (Range: 9^13^–72,284^41^; retrospective: n = 94,914, prospective: 4686) being carried out in a total of 93,344 patients over a 23-year period. A total of 2427 minor and 653 major complications were reported. Demographic data and procedural characteristics of individual studies are summarised in Supplementary Table S8. Most studies were single centre studies (n = 25) and six were multicentre. Procedures were performed by angiologists, vascular surgeons, radiologists, and cardiologists. Most studies were published by authors in the US (n = 16) or UK (n = 9, one multicentre UK/Greece^30^), but individual studies were from France (n = 1), Switzerland (n = 1), Poland (n = 2), Greece (n = 1, multicentre UK/Greece),^30^ Australia (n = 1), and Austria (n = 1). Most studies included recruitment of consecutive eligible patients. The patient populations differed between studies. All studies that reported sex included more male than female patients and the majority of studies included patients with CLTI and large proportion of co-morbidities (diabetes mellitus, arterial hypertension, CAD, CKD). The pooled estimate of technical success was 93% (95% CI 91–96%, reported in n = 16 studies). The rate of clinical success as defined by relief or substantial reduction of the preprocedural symptoms was not reported in any of the studies.

Twenty-three studies reported their standards for time to discharge which ranged from 1 h up to almost 8 h (Supplementary Table S8). These consisted typically of an initial time of flat bedrest (1–2 h) followed by an observation period and final clinical evaluation before discharge from the daycase unit by a healthcare professional. Some authors reported that earlier ambulation was allowed when a smaller sheath size was used (e.g., 4 F–4 h, 5 F–5 h, 6 F–6 h in^30^) and/or a vascular closure device used (e.g., 6 F–30 min, 8 F–90 min in^16^). In most studies sheath sizes of ≤6 F were used (0–42% ≥7 F).

Twenty-one studies reported that most procedures (89–100%) were performed via a common femoral access (Supplementary Table S6), with 0–42% bilateral access, and varying proportion of antegrade access (0–100% antegrade access). Two studies reported that in <1% brachial^20^^,^^30^ or retrograde popliteal^30^ access were additionally used. Three studies reported retrograde distal access cases^33^^,^^38^^,^^39^ with one exclusively reporting tibial access cases.^33^

Twenty-six studies reported about patient follow-up after discharge from the daycase unit. Twelve studies reported that patients had short-term (24–72 h) telephone or clinic consultations.^13^^,^^14^^,^^17^^,^^19^^,^^20^^,^^22^^,^^24^, ^25^, ^26^^,^^36^^,^^38^^,^^42^ Seven of these studies reported additional short term follow-up at 1–2 weeks,^38^ 10 days,^13^^,^^19^ and/or 30 days.^13^^,^^24^, ^25^, ^26^^,^^36^^,^^38^ Twelve studies reported the first follow-up at 1 week,^16^^,^^18^^,^^21^^,^^31^ 10 days,^32^ 2–4 weeks, 30 days,^30^^,^^33^, ^34^, ^35^^,^^37^ 2–4 weeks,^39^ or 4–6 weeks.^15^ Two studies reported follow-up appointments without specifying at what time.^23^^,^^27^

Some studies reported additional outcomes at 3 months,^16^^,^^18^^,^^31^, ^32^, ^33^ 6 months,^18^^,^^32^^,^^33^^,^^42^ and 12 months^32^^,^^33^ (not analysed).

### Selection criteria for daycase eligibility

Eighteen studies reported the criteria according to which patients were selected to be eligible for same day discharge procedures (Table 1). The most frequently reported criteria related to ensuring access to emergency care in case complications would occur including ‘responsible adult companion’ (94% of studies reporting eligibility criteria), the need to ‘stay close to hospital’ (71%), and maintain ‘telephone availability’ (59%). Studies also excluded unstable systemic cardiovascular conditions (29%) or severe life-threatening co-morbidities (*American Society of Anesthesiologists* [ASA] Classification >3, 35%), offset coagulation (known coagulopathy, raised INR, low platelets; 67%), and severe chronic kidney disease (53%). A further 47% of studies considered patients ineligible for daycase if there was a known contrast allergy or intolerance. Individual authors also excluded patients with high BMI (29%), electrolyte imbalance (18%), pregnancy (18%), poorly controlled hypertension (24%) or diabetes mellitus (24%), advanced age (12%), or CLTI (12%).

**Table 1:** Summary of eligibility criteria for daycase procedures as reported by authors.

### Definitions of major and minor complications

Twelve studies presented definitions of complications (Supplementary Table S5). Ten reported separate definitions of minor and major complication. Only four studies reported that definitions were based on classification systems of professional societies *CIRSE*,^32^ *SIR*,^30^^,^^43^ *or Society of Cardiovascular & Interventional Radiology*.^17^^,^^44^ One study referred to guidelines of the Food and Drug Administration.^25^ Authors’ definitions differed between studies. Definitions of minor complications included treatment-related adverse events requiring no further intervention including overnight hospitalization for observation only^22^^,^^30^^,^^32^^,^^38^; whereas major complications constituted those requiring further intervention with increase in the level of care and prolonged hospitalization. In some studies, hospitalisation per se was defined as a major complication.^15^^,^^36^

### Pooled estimation of complication incidence

**Fig. 2:**
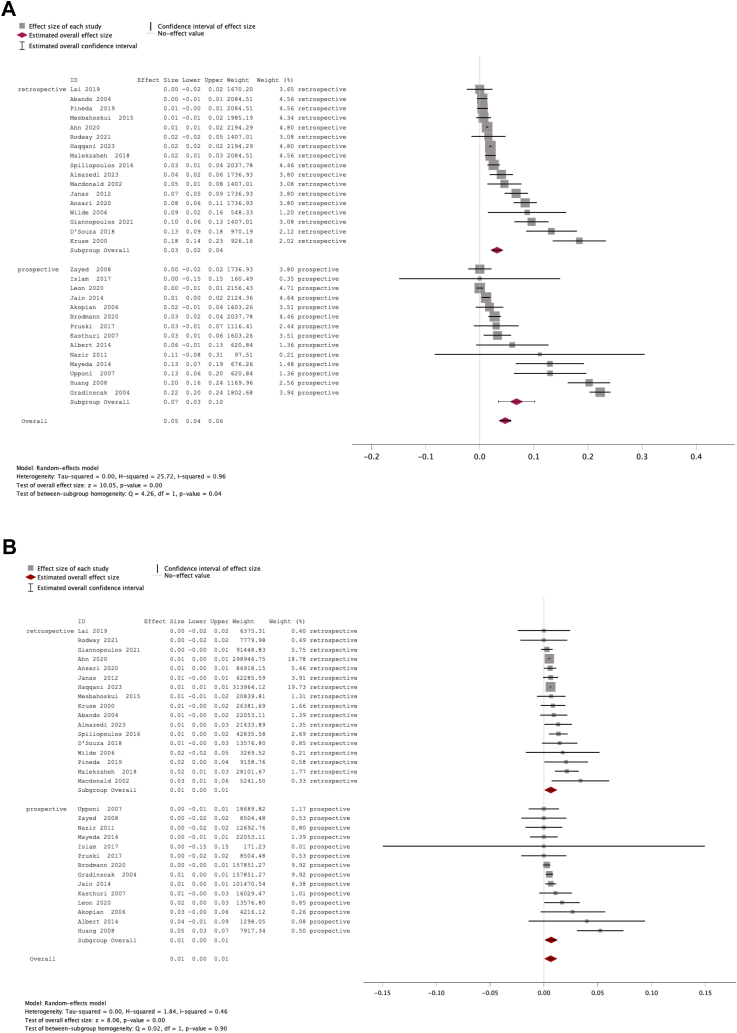
Forest plot showing (A) minor and (B) major complications in all 31 studies.

**Fig. 3:**
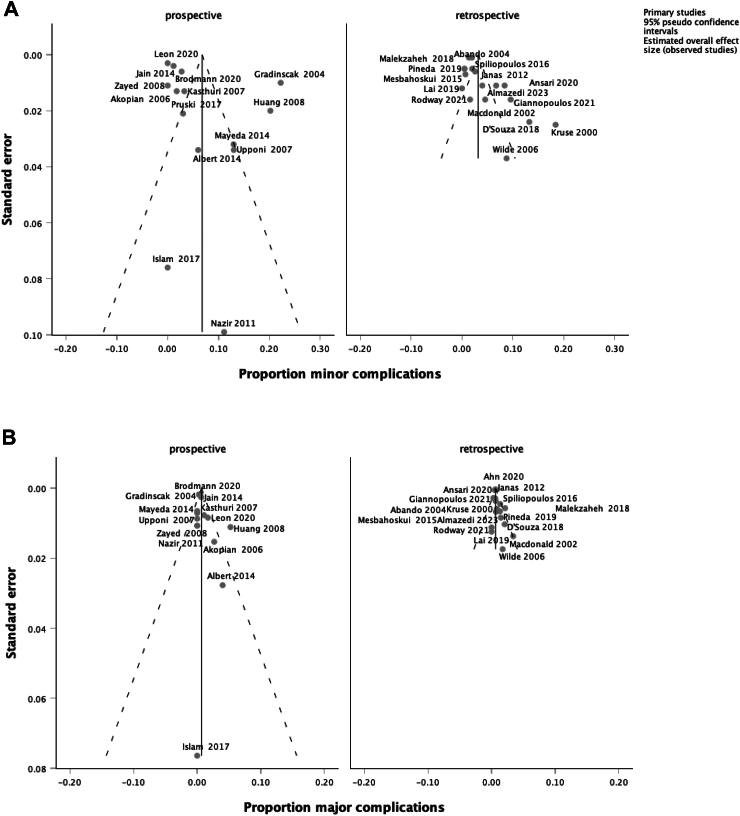
Funnel plot based (A) minor and (B) major complications in all 31 studies. Egger’s test p = 0.310 (A) and p = 0.079 (B).

In the first instance, we analysed minor and major complications in all 31 studies (Supplementary Table S7). In fourteen studies, minor and major complications were explicitly separately reported, and three studies reported that no complications occurred.^13^^,^^23^^,^^33^ In the remaining 14 studies, we adjudicated individual complications to major or minor based on clinical judgement applying the SIR criteria. The pooled incidence of minor and major complications in all studies were 4.7% (95% CI 3.8–5.6%; *I*^2^ = 96%) and 0.64% (95% CI 0.48–0.79%; *I*^2^ = 46%), respectively (Fig. 2, Fig. 3 and Table 2). As two large retrospective registry studies^35^^,^^41^ contributed the majority of cases, we performed a sensitivity analysis without these studies and this showed 5.7% (95% CI 4.02–7.40%, *I*^*2*^ = 96.2%) minor and 0.81% (95% CI 0.53–1.09%, *I*^*2*^ = 44.9%) major complications. We calculated major and minor complication rates separately in all retrospective and prospective studies. This showed that in retrospective studies minor complications were lower (3.2% [95% CI 2.4–3.9%], *I*^*2*^ = 92%) than in prospective studies (6.8% [95% CI 3.4–10.1%], *I*^*2*^ = 98%). Major complications were similar (retrospective: 0.65% [95% CI 0.50–1.9%], *I*^*2*^ = 38%; prospective: 0.68% [95% CI 0.23–1.1%], *I*^*2*^ = 54%). Twenty studies reported if complications were detected during the daycase visit or afterwards. The incidence of major complications occurring after discharge and leading to re-admission was 0.081% (95% CI −0.033 to 0.19%; *I*^2^ = 0%). In most studies, it was possible to re-assign complications according to the classification of CIRSE (n = 27 studies included) and SIR (n = 29 studies included).^11^^,^^12^ CIRSE 1–2 (minor) complications were 6.2% (95% CI 4.2–8.1%; *I*^2^ = 96%) and CIRSE 3–6 (major) complications were 0.50% (95% CI 0.34–0.66%; *I*^2^ = 3.3%). Based on SIR, minor complications were 6.2% (95% CI 4.3–8.0%; *I*^2^ = 96%) and major complications were 0.57% (95% CI 0.35–0.78%; *I*^2^ = 24%).

**Table 2:** Estimated incidence of complications.

	Effect size	95% CI		95% PI		*I*^2^
		Lower	Upper	Lower	Upper	
**Minor complications total (n = 31)**	**4.7%**	**3.8%**	**5.6%**	**0.24%**	**9.2%**	**96%**
Prospective studies (n = 14)	6.8%	3.4%	10.1%	−6.3%	19.9%	98%
Retospective studies (n = 17)	3.2%	2.4%	3.9%	0.55%	5.8%	92%
Minor complications (without studies reporting pain, n = 28)	2.7%	2.1%	3.3%	0.23%	5.1%	89%
CIRSE 1–2 minor (n = 27)	6.2%	4.2%	8.1%	−3.8%	16%	96%
SIR minor (n = 29)	6.2%	4.3%	8.0%	−3.7%	16.1%	96%
**Minor any bleeding**	**1.9%**	**1.4%**	**2.3%**	−0.21%	4.0%	**98%**
Minor puncture site haematoma/bruising/bleeding	1.6%	1.1%	2.0%	−0.39%	3.6%	98%
**Minor pain (n = 3)**	**3.8%**	**−1.0%**	**8.5%**	**−57%**	**65%**	**98%**
**Minor (perforation, dissection, embolism fixed during procedure)**	**0.064%**	**−0.024%**	**0.037%**	**−0.053%**	**0.066%**	70%
**Minor other and not specified**	**0.57%**	**0.30%**	**0.85%**	**−0.60%**	**1.75%**	**89%**
**Major complications total (n = 31)**	**0.64%**	**0.48%**	**0.79%**	**0.24%**	**1.03%**	**46%**
Prospective studies (n = 14)	0.68%	0.23%	1.1%	−0.50%	1.9%	54%
Retrospective studies (n = 17)	0.65%	0.50%	1.9%	0.34%	0.95%	38%
Major complications after discharge leading to re-admission (n = 20)	0.081%	−0.033%	0.19%	−0.041%	0.20%	0%
CIRSE 3–6 major (n = 27)	0.50%	0.34%	0.66%	0.27%	0.73%	3.3%
SIR major (n = 29)	0.57%	0.35%	0.78%	<0.0001%	1.1%	24%
**Major any bleeding**	**0.14%**	**0.053%**	**0.22%**	**−0.052%**	**0.32%**	**75%**
Major puncture site bleeding/haematoma	0.064%	0.0018%	0.13%	−0.091%	0.22%	68%
Major retroperitoneal bleeding/haematoma	0.000028%	−0.0019%	0.002%	−0.0020%	0.0020%	0%
Major pseudoaneurysm (surgery/thrombin)	0.000019%	−0.0019%	0.0019%	−0.0020%	0.0020%	0%
**Major any ischaemia/occlusion**	**0.28%**	**0.10%**	**0.45%**	**−0.37%**	**0.92%**	**93%**
Major occlusion (including flow limiting dissection)	0.030%	−0.011%	0.071%	−0.057%	0.11%	76%
Major embolus (including wire retention)	0.17%	0.018%	0.320%	−0.37%	0.71%	91%
Major thrombosis	0.00001%	−0.0019%	0.0019%	−0.0020%	0.0020%	0%
**Major infection access site (surgery)**	**0.000025%**	**−0.0018%**	**0.0019%**	**−0.0020%**	**0.0020%**	**0%**
**Major anaphylaxis/contrast**	**0.000018%**	**−0.0019%**	**0.0019%**	**−0.0020%**	**0.0020%**	**0%**
**Major systemic cardiovascular**	**0.000032%**	**−0.0019%**	**0.0019%**	**−0.0020%**	**0.0020%**	**0%**
**Major not specified**	**0.073%**	**−0.027%**	**0.17%**	**−0.25%**	**0.40%**	**69%**
**Death**	**0.00160%**	**−0.0018%**	**0.0021%**	**−0.0019%**	**0.0022%**	**0%**
**Conversion to hospitalised (n = 27)**	**1.6%**	**1.1%**	**2.2%**	**−0.55%**	**3.80%**	**82%**
**Re-admission after discharge (n = 23)**	**0.11%**	**0.095%**	**0.23%**	**−0.12%**	**0.25%**	**0%**
**Technical success (n = 15)**	**93%**	**91%**	**96%**	**85%**	**102%**	**97%**

CI = confidence interval, PI = prediction interval.

Minor complications were predominantly related to bleeding 1.9% (95% CI 1.4–2.3%, *I*^2^ = 98%) with the majority being small access site haematomas/bruising (1.6%) and a small proportion of pseudoaneurysms and arteriovenous fistulas that required no treatment. Only three studies reported pain as a minor complication^17^^,^^22^^,^^31^ and in these studies the pooled incidence of pain was 3.8%. As a sensitivity analysis, after removal of these three studies, the overall pooled incidence of minor complications in the remaining 28 studies decreased to 2.7% (95% CI 2.1–3.3%, *I*^2^ = 89%) indicating that reporting of minor pain greatly influenced the pooled estimate of minor complications.

Major complications were predominantly categorised as ischaemia/vessel occlusion (0.28%) and bleeding (0.14%). Ischaemia/occlusion was mostly related to embolus including wire retention (0.17%) and flow-limiting dissection (0.03%) and very rarely to thrombosis (<0.01%). Major bleeding complications were mostly at the access site (0.064%) and very rarely further specified as pseudoaneurysms or retroperitoneal. Anaphylaxis, severe access site infections and systemic cardiovascular complications were also very rare (<0.01%). Death was only reported in 0.0016% of cases.

On average, conversion from daycase to hospitalisation was 1.6% (95% CI 1.1–2.2%) while re-admissions after initial discharge from the daycase unit were 0.11% (95% CI 0.095–0.23%).

### Publication bias and sensitivity analysis according to Newcastle Ottawa scale categories

Supplementary Table S4 demonstrates the results of the assessment of bias using the *Newcastle Ottawa Scale*. Eight papers were deemed as poor quality,^16^^,^^25^^,^^27^^,^^34^, ^35^, ^36^^,^^42^^,^^43^ 18 as fair, and six as high quality. Within the selection domain, all except four appeared representative of patients requiring lower limb revascularisation as they were consecutive patients or registry data. Only six of the studies also reported complications of inpatients as controls along with the daycase outpatients (analysis shown below). As all procedures were preceded by clinical assessments and a diagnostic angiogram is the first step of all procedures, all studies were deemed to have adequately demonstrated that outcomes (complications) were absent pre-procedure. Within the comparability domain, only 17 studies reported eligibility criteria for daycases. Within the outcome category, all complications reported based on medical records or (unblinded) assessment by a healthcare professional. All studies were deemed to have long enough follow-up i.e., until discharge from daycase unit and this was completed in all included patients. Of note, the assessment of complications was mostly clinical (reported in n = 22 studies) with ultrasound performed if complications were suspected. Two studies^24^^,^^32^ reported that all patients received an ultrasound scan together with clinical assessment before discharge and one study that ultrasound was performed at 24 h.^36^ As some complications were initially self-reported by patients after discharge and only then added to medical records, we performed a sensitivity analysis on the subgroup of 20 studies that explicitly reported 30 day complication rates hypothetically capturing more complications than studies only evaluating the shorter timeframe until discharge. The minor complication rate in these studies was 2.8% (95% CI 1.9–3.7%, *I*^2^ = 88%) and major complication was 0.61% (95% CI 0.41–0.80%, *I*^2^ = 17%) which is similar or less then overall complication rates calculated in all 31 studies. As described above, the major complications occurring after discharge reported in 20 studies was 0.081% (95% CI −0.033 to 0.19%; *I*^2^ = 0%) suggesting that only very small proportion of major complications occur after discharge. The pooled incidence of minor complications of studies rated as good, fair, and poor were 6.0% (95% CI 1.9–10.1%; *I*^2^ = 93%), 6.6% (95% CI 3.9–9.2%; *I*^*2*^ = 97%), and 2.2% (95% CI 1.1–3.3%; *I*^2^ = 87%), respectively. The pooled incidence of major complications of studies rated as good, fair, and poor were 0.63% (95% CI 0.57–0.69%; *I*^2^ = 0%), 0.95% (95% CI 0.53–1.4%; *I*^2^ = 64%), and 0.52% (95% CI 0.43–0.62%; *I*^2^ = 0%), respectively.

Symmetrical Funnel plots (Fig. 3) and Egger’s test indicate low risk of publication bias for both minor and major complications (p = 0.310 and 0.079, respectively).

### Meta-regression analysis

**Fig. 4:**
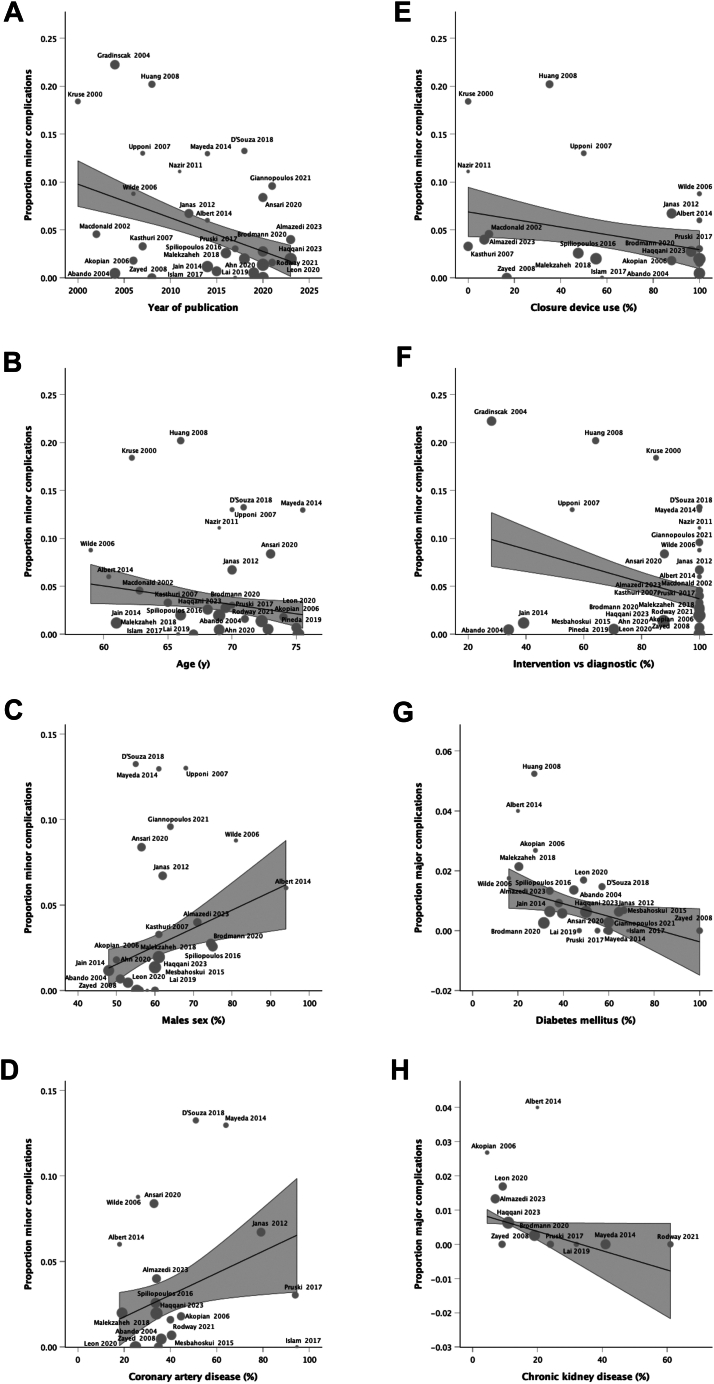
Bubble plot of meta-regression analyses showing (A) minor complications according to (A) publication year, (B) patient age, percentage of (C) male sex, (D) coronary artery disease (CAD), (E) vascular closure device, and (F) interventional procedures (remaining procedures were diagnostic). Major complications according to proportion of patients with (G) diabetes mellitus and (H) chronic kidney disease (all p < 0.05 Table 3; meta-regression prediction line with 95% confidence interval).

With the intention to identify sources of bias (in particular regarding minor complications), we performed univariate meta-regression analyses (Fig. 4, Table 3). Interestingly, minor complications significantly decreased over the analysed time period (−0.35%/year). Higher age (−0.19%/year), use of closure devices (−0.039%), and proportion of interventions (−0.086%) were associated with lower minor complications. Coronary artery disease (+0.064%) and male sex (+0.11%) were associated with higher minor complication rates. Major complications were significantly lower in patients with diabetes mellitus (−0.021%) and chronic kidney disease (−0.028%).

**Table 3:** Summary of univariate meta-regression analyses (B = unstandardised regression coefficient/effect estimate).

A	B	95% CI		p
		Lower	Upper	
Minor complications				
Year	−0.35%	−0.50%	−0.20%	**<0.0001**
Age (y)	−0.19%	−0.38%	−0.0090%	**0.041**
Male sex (%)	0.11%	0.032%	0.18%	**0.0072**
Chronic limb-threatening ischaemia (%)	0.00084%	−0.035%	0.037%	0.96
Coronary artery disease (%)	0.064%	0.0070%	0.12%	**0.030**
Hypertension (%)	0.039%	−0.017%	0.096%	0.16
Anticoagulants (%)	−0.15%	−0.31%	0.014%	0.68
Diabetes mellitus (%)	−0.033%	−0.092%	0.025%	0.24
Chronic kidney disease (%)	0.026%	−0.058%	0.11%	0.5
Current smoking (%)	0.00072%	−0.074%	0.075%	0.98
Sheath > 7 F (%)	0.0065%	−0.17%	0.19%	0.94
Closure device use (%)	−0.039%	−0.075%	−0.0037%	**0.032**
Intervention (%)	−0.086%	−0.13%	−0.0042%	**0.0004**
**B**	**B**	**Lower**	**Upper**	**p**
Major complications				
Year	0.011%	−0.038%	0.015%	0.39
Age (y)	−0.038%	−0.095%	0.018%	0.18
Male sex (%)	−0.0073%	−0.030%	0.015%	0.50
Chronic limb-threatening ischaemia (%)	−0.011%	−0.025%	0.0036%	0.13
Coronary artery disease (%)	−0.011%	−0.027%	0.0049%	0.16
Hypertension (%)	−0.011%	−0.022%	0.0006%	0.062
Anticoagulants (%)	0.016%	−0.024%	0.055%	0.38
Diabetes mellitus (%)	−0.021%	−0.041%	−0.0014%	**0.037**
Chronic kidney disease (%)	−0.028%	−0.056%	−0.0002%	**0.048**
Current smoking (%)	0.0097%	−0.0080%	0.027%	0.25
Sheath > 7 F (%)	−0.000062%	−0.041%	0.0041%	1.0
Closure device use (%)	−0.0066%	−0.017%	0.0032%	0.18
Intervention (%)	0.00041%	−0.0065%	0.0073%	0.90

*p* < 0.05 are in bold.

### Meta-analysis of complications between inpatients and outpatients

**Fig. 5:**
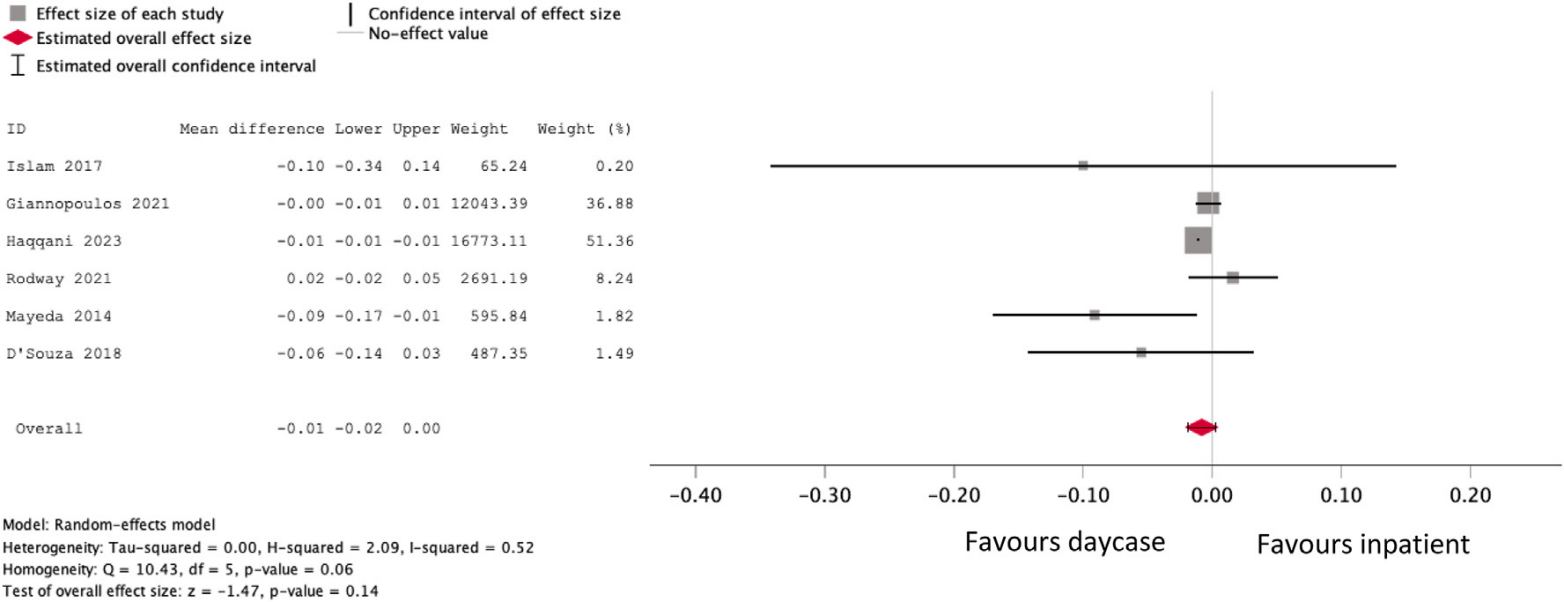
Forest plot comparing pooled incidence of total complications in patients undergoing daycase compared to inpatient angioplasty.

Six studies reported complications of angioplasties performed both as daycases (n = 73,023 procedures) and in inpatients (n = 60,099 procedures). As two of the studies only reported overall complication rates and did not distinguish between major and minor, we performed the analysis based on total complications (Fig. 5). This indicated that complications did not significantly differ between daycases and inpatients (pooled estimate if difference: −0.8% [95% CI −1.9 to 0.3%], *I*^2^ = 52%).

## Discussion

The major concern to be considered in the implementation of daycase-based endovascular procedures is patient safety. This is the first meta-analysis to evaluate the safety and efficacy of lower extremity daycase angioplasty. It shows a low rate of minor (4.7%) and major complications (0.64%) in mostly observational studies. Our data highlight that most complications arise from the access site, not the procedure itself, and most become apparent during the observation period. The pooled incidence of complications were well below the ‘acceptable’ thresholds for major vascular access (3%), systemic (1%) and catheter-induced complications (1%) proposed by the SIR.^45^^,^^46^ However, there was considerable heterogeneity between studies, in particular for minor complications (*I*^2^ = 96%). Only 11 of the studies provided definitions of complications and only 14 explicitly distinguished between major and minor complications (3 reported that no complication occurred). The reported range of minor complications ranged from zero^13^^,^^23^^,^^33^^,^^38^ to 22%.^17^ Only three studies reported pain as a complication and in these studies the incidence was 3.8% suggesting that this may be a source of heterogeneity. After removal of the studies reporting pain (sensitivity analysis), the heterogeneity and total minor complications rate decreased to 2.6% leaving access site bleeding the dominant cause of minor complications (1.8%). Nazir et al. have evaluated the severity of pain after angioplasty and showed that 63% had no pain, 31% had mild pain, and 5% moderate pain.^42^ While it may be argued if pain qualifies as a complication, it highlights that pain is a common symptom caused by the puncture site and also the endovascular procedures that needs to be addressed to deliver optimal treatment from a patient perspective. Major complications ranged from 0% to 3.4% and the reporting of major complications was less heterogeneous (*I*^2^ = 37%). Some studies considered overnight admission for observation as a major complication which does not align with *CIRSE* or *SIR* criteria. Re-adjudication of complications according to CIRSE 3–6 (0.49%) and SIR major (0.56%) criteria decreased the heterogeneity substantially (*I*^2^ = 3.8% and 25%) as both systems classify prolonged observation without additional treatments as minor. The dominant causes of major complications were limb ischaemia (0.27%; embolus, occlusion, thrombus) and bleeding (0.13%; puncture site, retroperitoneal, pseudoaneurysm). These complication rates appear lower than reported for coronary procedures. For instance, a recent RCT of 610 patients planned for treatment of coronary chronic total occlusion treatment reported a total of 2.0% (transradial) and 5.6% (transfemoral, p = 0.019) access site complications of which 1.6% and 3.9% were major bleeding and vascular complications requiring intervention.^47^ In the current analysis most studies predominantly employed femoral access with varying degrees of antegrade or retrograde approaches using rather ≤7F sheaths. However, one study exclusively reported about retrograde tibial approach without any complications.^33^ Similar to coronary procedures, the tibial approach may be a way forward to further reduce complications. Taken together, our data confirm that the safety of endovascular procedures largely hinges on puncture site problems highlighting the importance of careful access site management as a prerequisite for safe daycase angioplasties.

Eligibility criteria serve the purpose of maximising the safety and mitigating risks associated with the procedures by excluding patients with high-risk features (medical factors) and ensuring timely detection and access to treatment if complications occur after discharge (sociodemographic criteria). However, the choice of individual eligibility criteria is largely subjective and has not been prospectively validated in the context of daycase procedures. Some criteria are based on subjectively perceived or known risk factors of well-known complications.^48^ For instance, bleeding and thrombotic occlusions or thromboembolism are well-known and feared complications of arterial procedures. Therefore, abnormal coagulation very likely puts patients at increased risk in particular if bridging is required. The exact definitions of ineligible ‘coagulopathies’ and cut-off values for INR and platelets require further scrutiny. Several studies deemed patients with severe chronic kidney disease as ineligible for daycase angioplasty. Acute kidney injury after exposure to contrast medium is another feared complication that is more likely in patients with already impaired kidney function and systemic disease.^49^ Patients with CKD may require additional monitoring and therapy to prevent contrast-associated nephropathy and it may not be detected on the day of the procedure as it typically occurs 2–3 days after exposure. Therefore, hospital admission seems like a more sensible approach for patients with severe CKD. In addition, several studies reported that patients with severe or unstable cardiovascular conditions or life-threatening condition were ineligible for same day discharge procedures, some have defined this by ASA >3. A few studies also excluded patients with severe obesity, high age, not well controlled hypertension or diabetes mellitus, and previous contrast intolerance/allergy. While it has not been specifically investigated, all these factors likely put patients at increased risk for adverse events and the stress of a vascular procedure may trigger this. Hospital admission in these high-risk patients seems sensible. The data indicate that most complications occurred early during the observation period in the daycase unit. However, potentially life or limb-threatening complications could occur after discharge of the patients. To mitigate this, most studies explicitly required a ‘responsible adult companion’ to stay with patients overnight, to ‘stay close to hospital’, maintain ‘telephone availability’, and good comprehension, cooperation, and mobility.

While the eligibility criteria lead to patient selection and limit the generalisability of results, the studies included in this review have covered a broad range of patients across almost the entire spectrum of real-world patients with PAD, settings, and procedures. Most studies included patients with both CLTI and claudication, one study focused on repeated procedures in patients with diabetes mellitus and CLTI^23^ and two others included only patients with claudication.^13^^,^^18^ Janas found no difference in complications in patients younger and older than 70 years.^24^ Some studies only included retrograde chronic total occlusions of tibial arteries^33^^,^^38^ or atherectomy procedures^29^ but reported low complication rates indicating that even complex procedures can be performed safely with same day discharge. Eighteen studies reported if and how many closure devices were used. Only one study reported exclusively manual compression for haemostasis^20^ and one the use of a mechanical compression device,^42^ both with n = 0 *CIRSE* 3–6 complications. One study showed no difference in complications between manual compression and the use of Angioseal occlusion devices, but shorter time to haemostasis with Angioseal devices.^21^ Others only included patients with specific individual closure devices (Angioseal,^16^ Perclose,^19^ Mynx^25^). One study showed similar outcomes after 4F and 6F sheaths.^37^ Kruse showed that early discharge (≤4 h observation period) did not result in an increased readmission rate to the hospital.^14^ D’Souza showed that the efficacy and safety of outpatient endovascular tibial artery interventions between office and hospital settings were similar, with lower unplanned admission rates and better patency.^31^ In three UK studies, nurse-led daycase unit management was performed.^20^^,^^22^^,^^42^ One small randomised controlled trial compared same day discharge with overnight observation^13^ and five other studies reported complications in parallel groups of hospitalised patients along with daycase.^29^^,^^31^^,^^39^, ^40^, ^41^ Our meta-analysis including these 6 studies, showed no significant difference in complications between hospitalised vs daycase angioplasties. This however may be confounded by potential differences in characteristics of patients. Despite the fact that some individual studies focused on specific populations or addressed specific aspects in terms of setting and procedure, the low heterogeneity in particular of major complications supports the safety of daycase procedures in a large proportion of patients.

We attempted to explore the heterogeneity of complications with meta-regression analysis. This showed that the largest predictor of minor complications was the year the study was published suggesting that practise has improved over time. While some studies have excluded patients older than 80 years, in our analysis higher age was associated with lower minor complications. One feasible explanation maybe that older people are often frailer and interventionalists may prefer a minimalist approach with smaller sheaths and shorter procedure times to reduce the risk of potential serious complications, especially in a daycase setting. Coronary artery disease and male sex was associated with higher minor complications. It may be speculated that patients with CAD may be more frequently or more adherent to antithrombotic therapy. As expected,^48^ closure device use was associated with lower minor complications. Interventions (vs diagnostic procedures) did not predict higher complications but rather were associated with lower minor complications. This indicates that even diagnostic procedures require close attention to the access site at least as much as the interventions.

In terms of major complications, diabetes mellitus, and chronic kidney disease were associated with lower major complications. Again, interventionalists may chose a more minimalist approach or may be even more careful with the access in these patients as they are generally perceived to have more advanced disease and are at higher risk. Surprisingly, the presence of more complex disease such as CLTI and the use of larger sheaths (percentage >7 F) with assumed higher bleeding complication potential were no significant risk factors for complications which aligns with a previous study.^48^ This may be due to the overall low complication rate of daycase angioplasty and tendency to use smaller sheaths which in turn may be related to case selection. Many studies excluded high risk patients such as those with severe cardiac issues or socioeconomic factors for example socially isolated patients without overnight companion, not located within proximity of a hospital, those over 80 years, or severely overweight. While criteria such as this are a step towards formulating daycase inclusion criteria, the exclusion of too many factors may limit the population of patients for whom daycase revascularisation is applicable. Based on the eligibility criteria used in most studies, and in part informed by our meta-regression analysis, we present a draft set of medical, sociodemographic, and procedural criteria to be considered when planning daycase procedures (Table 4). Future studies are required to validate these criteria and evaluate if daycases can be performed with provision of risk control measures in patients currently deemed ‘not eligible’. Provision of trained ‘responsible companions’ with a car and telephone or electronic remote monitoring technology may be feasible options for socially isolated patients that would otherwise have to stay in the hospital for overnight observation.

**Table 4:** Proposed standard daycase eligibility criteria.

**Sociodemographic criteria**
Responsible adult present overnight
Stay close/within reasonable travelling distance to hospital overnight
Access to a telephone
Good comprehension of instructions and demonstrates cooperation/engagement with treatment and care recommendations and has mental capacity
No major immobility or contraindicating disability
**Medical**
Coagulation (no coagulopathies, International normalized ratio <1.5, platelets >75 × 10^9^/l, no bridging)
ASA 1–3; No unstable systemic cardiovascular condition
No severe kidney disease (eGFR > 30 ml/min/1.73 m^2^)
No contrast allergy, intolerance, severe allergies

Ambulatory procedures may require some rethinking: adaptation of care pathways and provision of resources that differ from inpatient procedures. The procedure planning process including assessment of risks and eligibility has to be completed before the admission and in particular safety aspects to prevent and manage complications at home and during transport need be considered. Clearly, patient choice plays an important role. Several studies confirmed high rates of patient acceptability.^13^^,^^19^^,^^20^^,^^22^ In one study, 98% of patients stated that they would be happy to undergo outpatient angioplasty or stenting again and only one patient preferred to undergo inpatient treatment in the future.^19^ However, up to 15% of patients may require hospital admission by choice or social reasons.^39^ This needs to be evaluated before admission to the daycase unit. In addition, procedure related factors need to be considered and if possible mitigated. The preferred use of vascular closure devices, if possible, may decrease ambulation time and help to decrease access site complications. Ultrasound guided puncture may help to avoid plaques and identify other contraindications for closure device use. In addition, the use of smaller sheaths facilitates faster ambulation and may decrease bleeding complication, but the latter has not been confirmed in previous studies.^37^^,^^48^ A daycase unit needs to accommodate sufficient time for post-procedural rest and observation. The time required has not been systematically studies but likely depends on the procedure and can likely be shorter when small size equipment and closure devices are used. This has important implications for vetting and staffing as the daycase procedures need to be completed earlier in the day to allow observation during normal working hours and prevent potential complications and patient transfer out of office hours. Finally, patients can only be discharged after clinical examination of the access site, evaluation of leg perfusion, and status of the patient ideally including ultrasound by the clinician in charge of the patient. Upon discharge, patients (and companions) need to be provided with an emergency telephone number, explicitly educated on potential complications and what to do if they occur. This underscores the need for ensuring that patients are collaborative and understand instructions. In addition, angioplasty is only part of the optimal medical management and also includes optimising medication for risk factor modification. Review of medication should be part of the daycase program and patients should receive a written treatment plan and be provided with new medication (i.e., platelet inhibitors and statins) on the day to ensure adherence, taking into account that patients should rather rest at home (with the responsible companion) and not be required to go to the general practitioner and pharmacy to get a prescription. As described previously,^50^^,^^51^ patients should leave the daycase unit with a follow-up appointment with a vascular specialist within 2–4 weeks to ensure the success of the procedure and adapted medication. Further follow-up visits including ankle brachial pressure index measurements and duplex ultrasound should be provided.^50^^,^^52^ The current data indicate that a short-term follow-up e.g., 24–72 h check-in phone call by a nurse is not strictly needed but may be useful for assurance in selected cases, patient experience of service, or when starting a new daycase service. Finally, and due to the heterogeneity of complications between studies that may be driven by site specific and unexpected factors, individual recording and monitoring of complications should be mandatory for quality monitoring and assurance.^9^

In conclusion, daycase procedures are a growing trend in modern healthcare systems, linked to technological improvements but also to shortage of economic and personal resources which needs to be carefully developed with a major focus on patient safety. Our data indicate that across a wide spectrum of carefully selected eligible PAD patients and procedures endovascular revascularisation can be performed with low complication and high technical success rates. Most complications arise from the puncture site and not the procedure itself highlighting the importance of optimal access site management including the use of occlusion devices if possible. The selection of suitable patients should be managed by an experienced vascular clinician and if in doubt prioritise on patient safety.

The large heterogeneity between studies warrants improvements in standardized documentation and monitoring of complications, technical, and clinical success. The complications should be documented, monitored in a standardised way (e.g., CIRSE, SIR criteria), and reviewed regularly. Patient engagement and compliance and mandatory follow-up appointments are critical to assure safety after discharge and clinical success comprehensively addressing other components of best medical therapy, respectively. Cost-effectiveness analyses are needed and should take costs for follow-up and ultimate outcome into the equation.

## Contributors

Conceptualization, ADR and CH; Data curation, LH, CH, ADR, PG, LM, JS; Formal analysis, LH, JH, SSS, CH; Investigation, na; Methodology, JH, LH, MBW, SSS, CH; Project administration, CH; Resources, CH; Software, CH; Supervision, LH, ADR, CH; Validation, OS, JM, VB, LB, MBW, CH; Visualization, LH and CH; Writing—original draft, CH; Writing—review & editing, LH, ADR, PG, LM, JS, OS, JM, VB, LB, MBW, SSS, JH, CH.

CH, PG, LM, LH, SSS have accessed and verified the data, and all authors were responsible for the decision to submit the manuscript.

## Data sharing statement

The data extracted from the original papers are available upon reasonable request pending a data sharing agreement.

## Declaration of interests

CH, JM and VB are members of the board of the European Society of Vascular Medicine. CH and OS are members of the nucleus of the European Society of Cardiology Working Group on Aorta and Peripheral Vascular Disease (CH treasurer and chairperson-elect, OS current chairperson). CH has received research funding from the European Partnership on Metrology, co-financed from European Union’s Horizon Europe Research and Innovation Programme and UK Research and Innovation, and Medical Research Council and honoraria for lectures by Bayer not related to the manuscript. CH declares being a council member and president-elect of Royal Society of Medicine, Vascular Medicine Council. All other authors declare no competing interests.
