# Supplementary material for: Safety and procedural success of daycase-based endovascular procedures in lower extremity arteries of patients with peripheral artery disease: a systematic review and meta-analysis
Source: eClinicalMedicine. 2024 Sep 5;75:102788. doi: 10.1016/j.eclinm.2024.102788 (PMC11406065; doi:10.1016/j.eclinm.2024.102788)
Supplement: Supplementary Figs. S1 and S2 and Tables S1–S8 [file mmc1.pdf]

Supplementary Table S1: Search criteria for OVID Medline and Embase

**Ovid MEDLINE**		118 articles
1	peripheral angioplasty.mp. [mp=title, abstract, heading word, drug trade name, original title, device manufacturer, drug manufacturer, device trade name, keyword heading word, floating subheading word, candidate term word]	
2	percutaneous transluminal angioplasty/	
3	lower limb angioplasty.mp. [mp=title, abstract, heading word, drug trade name, original title, device manufacturer, drug manufacturer, device trade name, keyword heading word, floating subheading word, candidate term word]	
4	1 or 2 or 3	
5	daycase.mp. [mp=title, abstract, heading word, drug trade name, original title, device manufacturer, drug manufacturer, device trade name, keyword heading word, floating subheading word, candidate term word]	
6	ambulatory surgery/	
7	day case.mp. [mp=title, abstract, heading word, drug trade name, original title, device manufacturer, drug manufacturer, device trade name, keyword heading word, floating subheading word, candidate term word]	
8	same day.mp. [mp=title, abstract, heading word, drug trade name, original title, device manufacturer, drug manufacturer, device trade name, keyword heading word, floating subheading word, candidate term word]	
9	outpatient.mp. [mp=title, abstract, heading word, drug trade name, original title, device manufacturer, drug manufacturer, device trade name, keyword heading word, floating subheading word, candidate term word]	
10	outpatient/ or outpatient department/	
11	5 or 6 or 7 or 8 or 9 or 10	
12	peripheral artery disease.mp. [mp=title, abstract, heading word, drug trade name, original title, device manufacturer, drug manufacturer, device trade name, keyword heading word, floating subheading word, candidate term word]	
13	peripheral occlusive artery disease/	
14	intermittent limb claudication.mp. [mp=title, abstract, heading word, drug trade name, original title, device manufacturer, drug manufacturer, device trade name, keyword heading word, floating subheading word, candidate term word]	
15	intermittent claudication/	

16	chronic limb threatening isch*emia.mp. [mp=title, abstract, heading word, drug trade name, original title, device manufacturer, drug manufacturer, device trade name, keyword heading word, floating subheading word, candidate term word]
17	critical limb ischemia/
18	12 or 13 or 14 or 15 or 16 or 17
19	4 and 11 and 18

Supplementary Table S2: Eligibility Criteria

Inclusion criteria	Exclusion criteria
• Any published study reporting the complications associated with angioplasty procedures in PAD patients as a day case surgery.<br>• Randomised or non-randomised cohort studies<br>• At least 5 participants of any age<br>• Reports complications associated with patients<br>• Majority regarding lower limb (angioplasty) procedure	• Not written in English<br>• Not original research e.g. systematic reviews, review articles, protocols, conference abstracts, editorials, or book chapters<br>• Case reports and case series<br>• Studies with non-stratified complications e.g. only lists that complications were present without detail

Supplementary Table S3. Definitions of complication classification systems. [1,2]

A. ***Cardiovascular and Interventional Radiology Society Europe (CIRSE)***

**Minor Complications**
1. Complication during the procedure which could be solved within the same session; no additional therapy, no post-procedure sequelae, no deviation from the normal post- therapeutic course
2. Prolonged observation including overnight stay (as a deviation from the normal post-therapeutic course <48 h); no additional postprocedure therapy, no postprocedure sequelae

**Major Complications**
3. Additional postprocedure therapy or prolonged hospital stay (>48 h) required; no postprocedure sequelae
4. Complication causing a permanent mild sequelae (resuming work and independent living)
5. Complication causing a permanent severe sequelae (requiring ongoing assistance in daily life)
6. Death

B. ***Society of Interventional Radiology*** **(SIR)**

**Minor Complications**
A. No therapy, no consequence, or
B. Nominal therapy, no consequence; includes overnight admission for observation only.

**Major Complications**
C. Require therapy, minor hospitalization (<48 h),
D. Require major therapy, un- planned increase in level of care, pro- longed hospitalization (<48 h),
E. Have permanent adverse sequelae, or
F. Result in death.

Supplementary Table S4: Newcastle-Ottawa Quality Assessment Form

**Selection**	
Representativeness of exposed cohort	Truly representative of daycase angioplasty patients (consecutive patients, population based registry) (*)
	Somewhat representative (*)
	only selected group of patients
	no description of derivation of cohort
Selection of non intervention cohort	Hospitalised patients (non-intervention group) drawn from same community as intervention cohort also reported (*)
	Drawn from different source
	No description of the derivation of the non-intervention cohort
Ascertainment of exposure	Health records (*)
	Prospective inclusion (*)
	No description
Outcome of interest was not present at start of study	Yes (*)
	No
**Comparability**	
Comparability of cohorts on basis of design or analysis	Definition of daycase criteria provided (*)
	Study controls for additional factors (*)
**Outcome**	
Assessment of outcomes	Independent blind assessment (*)
	Prospective assessment by healthcare professional at discharge (*)
	Retrospective based on healthcare records (*)
Was follow-up long enough for outcomes to occur	Yes (30 day) *
	Yes (24-72h follow-up) *
	Yes (before discharge) *
	No
Adequacy of follow up of cohorts	Follow-up performed (phone call/visit) *
	Before discharge (*)

Thresholds for converting the Newcastle-Ottawa scales:
Good quality: 3 or 4 stars in selection domain AND 1 or 2 stars in comparability domain AND 2 or 3 stars in outcome/exposure domain
Fair quality: 2 stars in selection domain AND 1 or 2 stars in comparability domain AND 2 or 3 stars in outcome/exposure domain
Poor quality: 0 or 1 star in selection domain OR 0 stars in comparability domain OR 0 or 1 stars in outcome/exposure domain

Supplementary Table S5. Newcastle Ottawa Scale grading system of each study to assess selection and outcome bias.

	1. Selection				2. Comparability		3. Outcome			Total score	Quality score
	Representativeness of exposed cohort	Selection of non intervention cohort (hosptalised)	Ascertainment of exposure	Outcome of interest was not present at start of study	Definition daycase eligibility	Additional factors	Assessment of outcome	Was follow up long enough for outcome (until discharge)?	Adquacy of follow up of cohorts (at discharge)		
**Kruse 2000**	*	-	*	*	*	*	*	*	*	8	high quality
**Macdonald 2002**	*	-	*	*	*	-	*	*	*	7	fair quality
**Abando 2004**	*	-	*	*	-	-	*	*	*	6	poor quality
**Gradinscak 2004**	*	-	*	*	*	-	*	*	*	7	fair quality
**Akopian 2006**	*	-	*	*	*	-	*	*	*	7	fair quality
**Wilde 2006**	*	-	*	*	*	-	*	*	*	7	fair quality
**Kasthuri 2007**	*	-	*	*	*	-	*	*	*	7	fair quality
**Upponi 2007**	*	-	*	*	-	*	*	*	*	7	fair quality
**Huang 2008**	*	-	*	*	*	-	*	*	*	7	fair quality
**Zayed 2008**	*	-	*	*	*	*	*	*	*	8	high quality
**Nazir 2011**	*	-	*	*	-	-	*	*	*	6	poor quality
**Janas 2012**	*	-	*	*	*	-	*	*	*	7	fair quality
**Albert 2014**	*	-	*	*	*	-	*	*	*	7	fair quality
**Jain 2014**	*	-	*	*	-	-	*	*	*	6	poor quality
**Mayeda 2014**	-	*	*	*	-	*	*	*	*	7	fair quality
**Mesbah Oskui 2015**	*	-	*	*	-	-	*	*	*	6	fair quality
**Spiliopoulos 2016**	*	-	*	*	*	-	*	*	*	7	fair quality
**Islam 2018**	-	*	*	*	*	*	*	*	*	8	high quality
**Pruski 2017**	*	-	*	*	-	-	*	*	*	6	poor quality
**D'Souza 2018**	*	*	*	*	-	*	*	*	*	8	high quality
**Malekzadeh 2018**	*	-	*	*	*	-	*	*	*	7	fair quality
**Lai 2019**	*	-	*	*	*	-	*	*	*	7	fair quality
**Pineda 2019**	-	-	*	*	-	-	*	*	*	5	poor quality
**Ahn 2020**	*	-	*	*	-	-	*	*	*	6	poor quality
**Ansari 2020**	-	-	*	*	-	-	*	*	*	5	poor quality
**Brodmann 2020**	-	-	*	*	*	*	*	*	*	7	fair quality
**Leon 2020**	*	-	*	*	*	-	*	*	*	7	fair quality
**Rodway 2021**	*	*	*	*	*	*	*	*	*	9	high quality
**Giannopoulos 2021**	-	*	*	*	-	*	*	*	*	7	fair quality
**Almazedi 2023**	*	-	*	*	-	-	*	*	*	6	poor quality
**Haqqani 2023**	*	*	*	*	-	*	*	*	*	8	high quality

Supplementary Table S6: Overview of access characteristics

	Common femoral access	Brachial artery	Popliteal artery	Tibial arteries	Bilateral access	Antegrade access	Crossover prodedure
Kruse 2000	89%				22%		
Macdonald 2002	"usually"						
Abando 2004	100%						
Gradinscak 2004							
Akopian 2006	100%						
Wilde 2006	100%				42%	14%	
Kasthuri 2007	99%	<1%			21%	45%	
Upponi 2007	100%				0%	0%	
Huang 2008	100%				8%	26%	
Zayed 2008	100%						
Nazir 2011	100%					67%	
Janas 2012	100%					82%	
Albert 2014	100%					6%	72%
Jain 2014	"almost always"						
Mayeda 2014							
Mesbah Oskui 2015							
Spiliopoulos 2016	99%	<1%	<1%			68%	
Islam 2018	100%					0%	
Pruski 2017	100%				0%	100%	0%
D'Souza 2018							
Malekzadeh 2018	100%				12%		
Lai 2019				100%			
Pineda 2019							
Ahn 2020							
Ansari 2020	100%						
Brodmann 2020	100%				1%	71%	
Leon 2020	100%			6%			
Rodway 2021	100%			3%		75%	25%
Giannopoulos 2021							
Almazedi 2023					21%		
Haqqani 2023							

Supplementary Table S7: Summary of complication definitions

	Definition provided (Y/N)	Report minor and major complications	Minor definition	Major definition
Kruse 2000	Y	Y	Not requiring hospitalization. In the case of minor haematomas, no distinction was made between the size of the hematoma.	Those that required the patient to be hospitalized. A hematoma that required a transfusion or admission as part of the treatment
Macdonald 2002	Y	Y	No sequelae: they possibly required normal therapy only or a short hospital stay for observation (generally overnight)	Admission to hospital for therapy, unplanned increase in the level of care, prolonged hospitalization, permanent adverse sequelae, death
Abando 2004	(Y)	Y	Complications including infection requiring antibiotic administration, pseudoaneurysm formation, or vessel compromise were recorded. Bleeding episodes causing a hematoma greater than 6 cm and a decrease in hemoglobin of more than 1 g, as well as those requiring transfusion or necessitating operation, were noted.	
Gradinscak 2004	(Y)	Y	Complications including infection requiring antibiotic administration, pseudoaneurysm formation, or vessel compromise were recorded. Bleeding episodes causing a hematoma greater than 6 cm and a decrease in hemoglobin of more than 1 g, as well as those requiring transfusion or necessitating operation, were noted. (SCVIR, Singh 2002)	
Akopian 2006	N	Y		
Wilde 2006	N	N		
Kasthuri 2007	N	N		
Upponi 2007	Y	Y	Haematoma size <6cm	Requiring further percutaneous or surgical intervention
Huang 2008	Y	Y	No permanent sequelae, and required only nominal therapy or a short hospital stay for observation	Any complication that required hospital admission for surgery or medical therapy, prolonged hospitalization or caused permanent adverse sequelae or death
Zayed 2008	N	N	(no complications observed)	
Nazir 2011	N	Y	Haematoma <5 cm	
Janas 2012	Y	Y		30-day major vascular complications - retroperitoneal bleeding, pseudoaneurysm, AV fistula, haematoma.
Albert 2014	N	N		
Jain 2014	N	N		
Mayeda 2014	N	N		
Mesbahoskui 2015	N	N		
Spiliopoulos 2016	Y	Y	(i) Requiring no therapy and no consequence, or (ii) those requiring nominal therapy but with no consequence, including overnight admission for observation only.	According to the SIR reporting standards as those (i) requiring therapy, minor hospitalization (48 h), (ii) requiring major therapy, unplanned increase in level of care, prolonged hospitalization (48 h), (iii) have permanent adverse sequelae, or (iv) resulted in death
Islam 2018	N	N	(no complications observed)	
Pruski 2017	Y	Y	Access site hematoma >6 cm, pseudoaneurysm resolved with thrombin injection or not requiring treatment, ipsilateral AV fistula, ipsilateral deep vein thrombosis (DVT), ipsilateral lower extremity arterial emboli, late access site–related bleeding	Access site–related surgical or vascular repair, permanent access site–related nerve injury, access site–related bleeding requiring transfusion, new ipsilateral lower extremity ischemia requiring surgical intervention, infection (FDA, Scheinert 2012)
D'Souza 2018	N	N		
Malekzadeh 2018	Y	Y	CIRSE classification (1-2)	CIRSE classification (3-6); additional post-procedure therapy to death
Lai 2019	N	N	(no complications observed)	
Pineda 2019	N	N		
Ahn 2020	N	N		
Ansari 2020	Y	Y	Minor complications were those treated within the facility with no sequelae, including minor perforation, distal embolization, allergic reactions, flow limiting dissection, and arteriovenous fistula formation.	major complications were those requiring inpatient hospitalization and included bleeding and wire retention. Additional criteria for major bleeding was a drop in hemoglobin of >3 g/dl and/or requiring transfusion.
Brodmann 2020	N	N		
Leon 2020	Y	N	Events that occurred during the entire stay of the patient at our OBL requiring further treatment such as the need for hospital transfer for either observation, blood transfusion or surgical or further endovascular intervention.	
Rodway 2021	N	N		
Giannopoulos 2021	N	N		
Almozedi 2023	Y	Y	Complication classification was according to the Society of Interventional Radiology reporting standards, with minor complications defined as (A) those necessitating no therapy and having no consequence, or (B) those necessitating nominal therapy and having no conse- quence except overnight admission for observation only.	Major complications were defined as (C) those necessitating minor therapy with <48 h of hospitalisation, (D) those necessitating major therapy with an unplanned increase in the level of care and hospitalisation >48 h, (E) resulting in permanent adverse sequelae, or (F) resulting in death.
Haqqani 2023	N	N		

Supplementary Table S8: Summary of studies included.

				Time to discharge (h)	30 day outcome	Follow-up	Country	Procedures (n)	Minor complications [n]	Major complications [n]	CIRSE 1-2 [n]	CIRSE 3-6 [n]	Conversion to hospitalisation [n]	Re-admission after discharge [n]	Age [yrs]	Male sex	Chronic limb-threatening ischaemia	Diabetes mellitus	Arterial hypertension	Warfarin/(N)OAC	Coronary artery disease	Chronic kidney disease	Dialysis	Smoking current	Smoking current and past	≥7F sheath	Closure device used	Angioplasty	Technical success
Kruse 2000	Retrospective	Single centre	Consecutive patients	2-6	-	24 h phone	US	239	44 (18.4%)	2 (0.8%)	46 (19.2%)	0 (0.0%)	2 (0.8%)	0 (0.0%)	62		15%			11%						8%	0%	85%	
Macdonald 2002	Retrospective	Single centre	Consecutive patients	3-5	•	4-6 wk clinic	UK	176	8 (4.5%)	6 (3.4%)	8 (4.5%)	6 (3.4%)	12 (6.8%)	6 (3.4%)	63					1%							39%	100%	
Abando 2004	Retrospective	Single centre	(Consecutive patients)	1-3	•	1, 4, 6, 12 wk clinic	US	218	1 (0.5%)	2 (0.9%)	1 (0.5%)	2 (0.9%)	1 (0.5%)	1 (0.5%)	69	53%	4%	38%	36%	24%	36%			44%	44%	13%	100%	35%	
Gradinscak 2004	Prospective	Single centre	Consecutive patients	2-6	-	24-48 h phone	AUS	1,569	349 (22.2%)	8 (0.5%)	349 (22.2%)	8 (0.5%)																28%	
Akopian 2006	Prospective	Single centre	Consecutive patients	3-4	•	1, 6 wk; 3, 6 mo clinic	US	112	2 (1.8%)	3 (2.7%)	4 (3.6%)	1 (0.9%)	9 (8.0%)	0 (0.0%)	74	50%	0%	28%	79%		45%	5%		54%	55%	7%	82%	100%	
Wilde 2006	Retrospective	Single centre	Consecutive patients	1.5 (median)	-	24 h phone, 10d clinic	UK	57	5 (8.8%)	1 (1.8%)	5 (8.8%)	1 (1.8%)	6 (10.5%)	0 (0.0%)	59	81%		16%	44%		26%					26%	100%	100%	
Kasthuri 2007	Prospective	Single centre	Consecutive patients	5	-	24 h phone	UK	183	6 (3.3%)	2 (1.1%)	6 (3.3%)	2 (1.1%)	2 (1.1%)	1 (0.5%)	65	61%				0%						5%	0%	100%	
Upponi 2007	Prospective	Single centre	Consecutive patients	2-3	•	1 wk phone, 6 wk clinic	UK	100	13 (13.0%)	0 (0.0%)	13 (13.0%)	0 (0.0%)	0 (0.0%)	0 (0.0%)	70	68%										2%	50%	56%	
Huang 2008	Prospective	Single centre	Consecutive patients	1-6	-	24h phone	UK	401	81 (20.2%)	21 (5.2%)	98 (24.4%)	4 (1.0%)	21 (5.2%)	1 (0.2%)	66			27%									35%	64%	
Zayed 2008	Prospective	Single centre	Consecutive patients	2-4	-	"clinic appointment"	UK	66	0 (0.0%)	0 (0.0%)	0 (0.0%)	0 (0.0%)	0 (0.0%)	0 (0.0%)	67	56%	100%	100%	67%		35%	9%		35%	35%		17%	100%	96%
Nazir 2011	Prospective	Single centre	Consecutive patients	4	•	24 h phone, 6 mo phone	UK	81	9 (11.1%)	0 (0.0%)	9 (11.1%)	0 (0.0%)	0 (0.0%)	0 (0.0%)	69											0%	0%	100%	
Janas 2012	Retrospective	Single centre	Consecutive patients	3-5	•	24 h phone/clinic, 30 d clinic	PL	477	32 (6.7%)	3 (0.6%)	33 (6.9%)	2 (0.4%)	3 (0.6%)		70	62%	20%	64%	95%		79%		22%	20%	20%	16%	88%	100%	93%
Albert 2014	Prospective	Single centre	Consecutive patients	4	•	24 h phone, 30 d clinic	F	50	3 (6.0%)	2 (4.0%)	3 (6.0%)	2 (4.0%)	8 (16.0%)	0 (0.0%)	60	94%	8%	20%	52%	6%	18%	40%		54%	84%		100%	100%	94%
Jain 2014	Prospective	Single centre	Consecutive patients		•	"clinic follow-up"	US	939	11 (1.2%)	6 (0.6%)	11 (1.2%)	6 (0.6%)	6 (0.6%)	1 (0.1%)	61	48%	23%	34%	62%					16%	16%			39%	
Mayeda 2014	Prospective	Mulit centre	Registry		-		US	108	14 (13.0%)	0 (0.0%)	14 (13.0%)	0 (0.0%)			76	61%	23%	60%	88%		64%	41%			75%			100%	93%
Mesbah Oskui 2015	Retrospective	Single centre	Consecutive patients	4	-		US	148	1 (0.7%)	1 (0.7%)	1 (0.7%)	1 (0.7%)	1 (0.7%)		75	51%	25%	67%	95%	15%	41%		16%	6%	35%			100%	82%
Spiliopoulos 2016	Retrospective	Mulit centre	Consecutive patients	4-6	•	30 day records	GR/UK	662	17 (2.6%)	9 (1.4%)	24 (3.6%)	2 (0.3%)	30 (4.5%)	3 (0.5%)	68	74%	25%	45%	54%	36%	34%		12%		36%	4%	48%	100%	98%
Islam 2018	Prospective/RCT	Single centre		2-4	•	48-72 h, 10d phone, 30d records	US	9	0 (0.0%)	0 (0.0%)	0 (0.0%)	0 (0.0%)	0 (0.0%)	0 (0.0%)	66	58%	0%	68%	100%		95%			42%	42%	32%	58%	100%	100%
Pruski 2017	Prospective	Single centre	Consecutive patients	4.8 (mean)	•	24 h, 30 d phone/clinic	PL	66	2 (3.0%)	0 (0.0%)	0 (0.0%)	2 (3.0%)	0 (0.0%)	0 (0.0%)	70			59%	92%		94%	24%	17%		62%	20%	100%	100%	95%
D'Souza 2018	Retrospective	Single centre	Consecutive patients		•	1, 3, 6, 12 wk clinic	US	204	27 (13.2%)	3 (1.5%)	27 (13.2%)	3 (1.5%)	27 (13.2%)	2 (1.0%)	71	55%	77%	57%	91%		51%			57%	57%			100%	
Malekzadeh 2018	Retrospective	Single centre	Consecutive patients	6	-	10 d, 3, 6, 12 mo records	CH	654	13 (2.0%)	14 (2.1%)	13 (2.0%)	14 (2.1%)	12 (1.8%)	4 (0.6%)	66	61%	9%	20%	55%		19%			45%	45%	4%	55%	100%	98%
Lai 2019	Retrospective	Single centre	Consecutive patients		•	30 d, 3, 6, 9, 12 mo records	US	57	0 (0.0%)	0 (0.0%)	0 (0.0%)	0 (0.0%)	0 (0.0%)	0 (0.0%)	67	60%	34%	47%	75%			32%	9%	53%	83%			100%	94%
Pineda 2019	Retrospective	Single centre		2-6	•	30 d records	US	193	1 (0.5%)	4 (2.1%)	5 (2.6%)	0 (0.0%)	5 (2.6%)	0 (0.0%)	73		42%											70%	
Ahn 2020	Retrospective	Mulit centre	Registry		•	30 d records	US	18,134	246 (1.4%)	92 (0.5%)			112 (0.6%)		72	60%	30%								65%			88%	
Ansari 2020	Retrospective	Single centre			•	48-72 h, 30 d clinic	US	692	58 (8.4%)	4 (0.6%)			4 (0.6%)	0 (0.0%)	73	57%	43%	40%	49%		33%	8%	9%		32%			88%	
Brodmann 2020	Prospective	Mulit centre		7.8 (mean)	•	30 d phone/clinic	A	765	21 (2.7%)	2 (0.3%)	21 (2.7%)	2 (0.3%)	38 (5.0%)		70	75%		31%	80%			19%			76%	0%	96%	100%	99%
Leon 2020	Prospective	Single centre	Consecutive patients	3	•	24-48 h phone, 1-2 wk clinic, 30 d records	US	237	0 (0.0%)	4 (1.7%)	5 (2.1%)	0 (0.0%)	4 (1.7%)	0 (0.0%)	75	55%		49%	62%		25%	9%						100%	84%
Rodway 2021	Retrospective	Single centre	Consecutive patients	4	•	2-4 wk clinic	UK	63	1 (1.6%)	0 (0.0%)	1 (1.6%)	0 (0.0%)	0 (0.0%)	0 (0.0%)	71		84%	55%	76%	24%	40%	61%	0%	17%	98%	0%	100%	100%	97%
Giannopoulos 2021	Retrospective	Multi centre	Registry		-		US	355	34 (9.6%)	1 (0.3%)						64%	52%	60%	95%	6%				20%	69%			100%	83%
Almazedi 2023	Retrospective	Single centre	Consecutive patients	3	•		US	301	12 (4.0%)	4 (1.3%)	12 (4.0%)	4 (1.3%)	7 (2.3%)	2 (0.7%)		71%	43%	34%	49%		34%	7%			83%	6%	7%	100%	96%
Haqqani 2023	Retrospective	Multi centre	Registry		-		US	72,284	1416 (2.0%)	459 (0.6%)					69	61%	44%	50%	88%	17%	34%	11%	6%	46%	39%	22%	100%	100%	93%

SUPPLEMENTARY FIGURE LEGENDS

Supplementary Figure S1: Forrest plot of 27 studies with available complications according to CIRSE criteria.[1] (A) Minor complications (CIRSE 1-2) overall: 5.0% (95%CI 3.4-6.6%), I^2*=96%%;* retrospective (n=17): 7.0% (95%CI 3.4-10.6%), I^2*=89%;* prospective (n=14): 4.6% (95%CI 2.7-6.6%), I^2*=98%.* (B) Major complications (CIRSE 3-6) overall: 0.50% (95%CI 0.34-0.66%), I^2*=3.3%;* retrospective (n=17): 0.7% (95%CI 0.34-1.06%), I^2=36%; prospective (n=14): 0.46% (95%CI 0.27-0.66%), I^2*=0%*

Supplementary Figure S2: Funnel plots of data shown in Supplementary Figure 1.

A

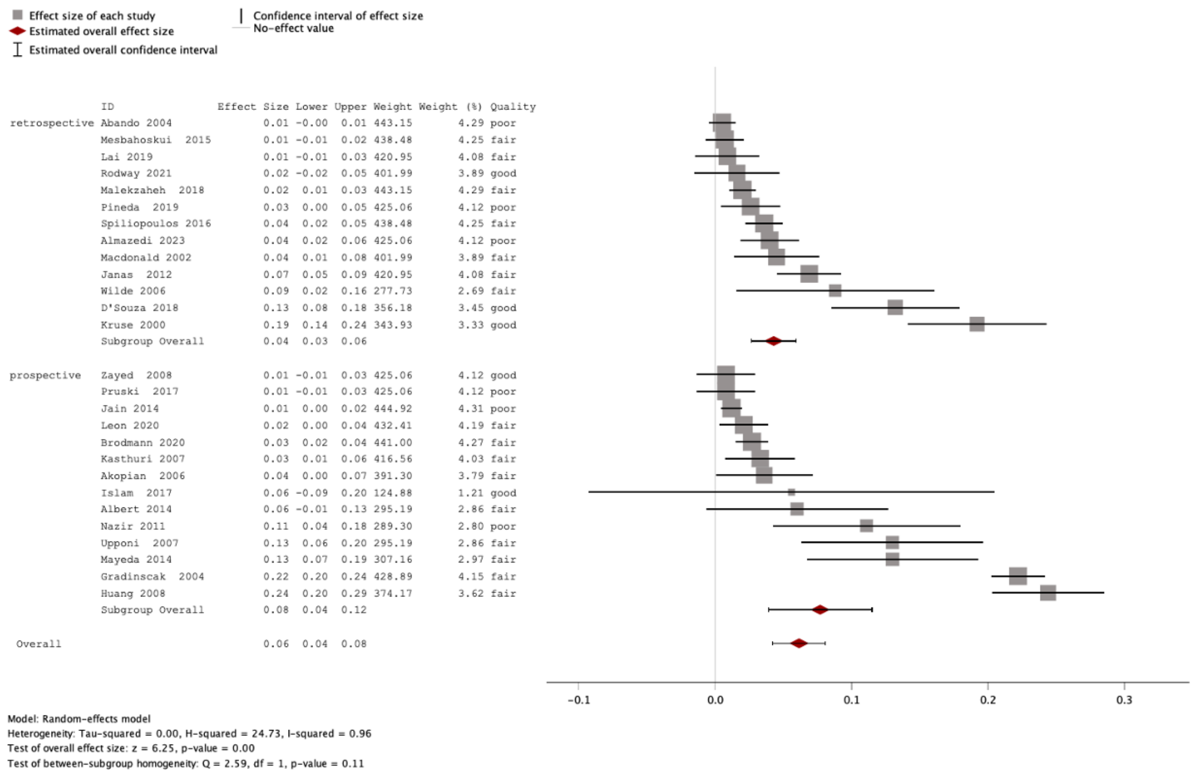

B

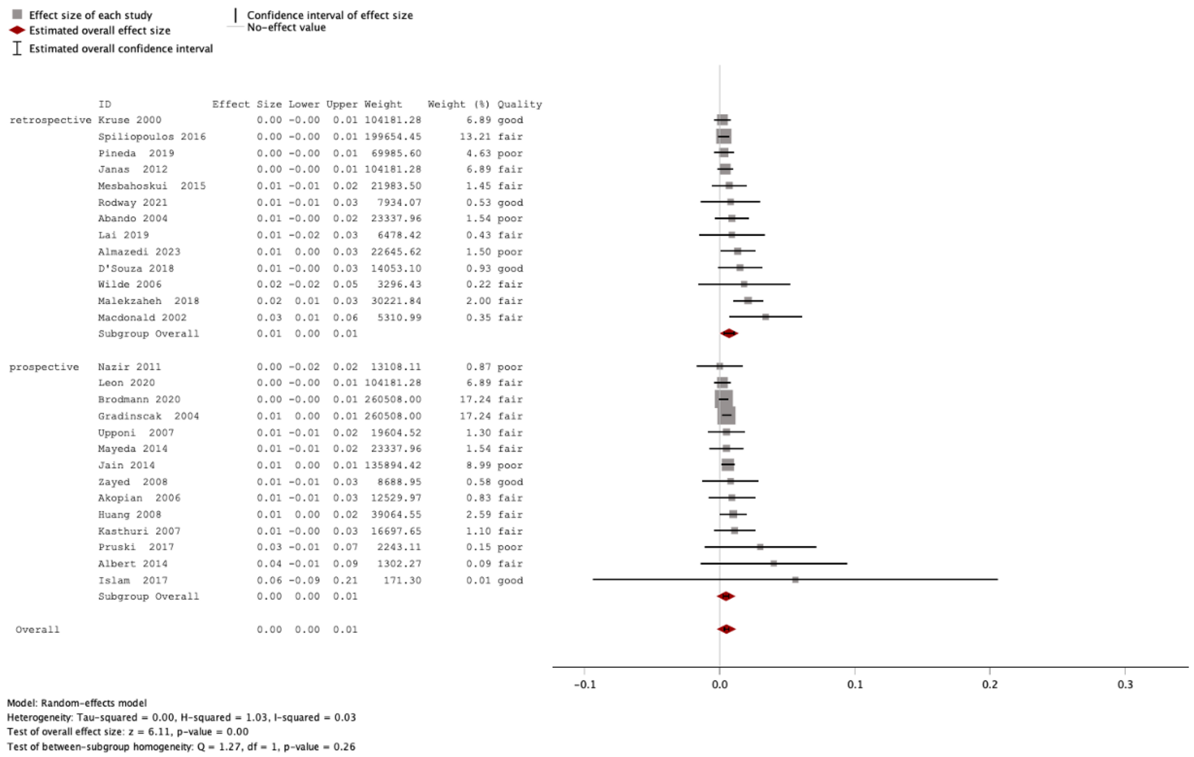

Supplementary Figure S1

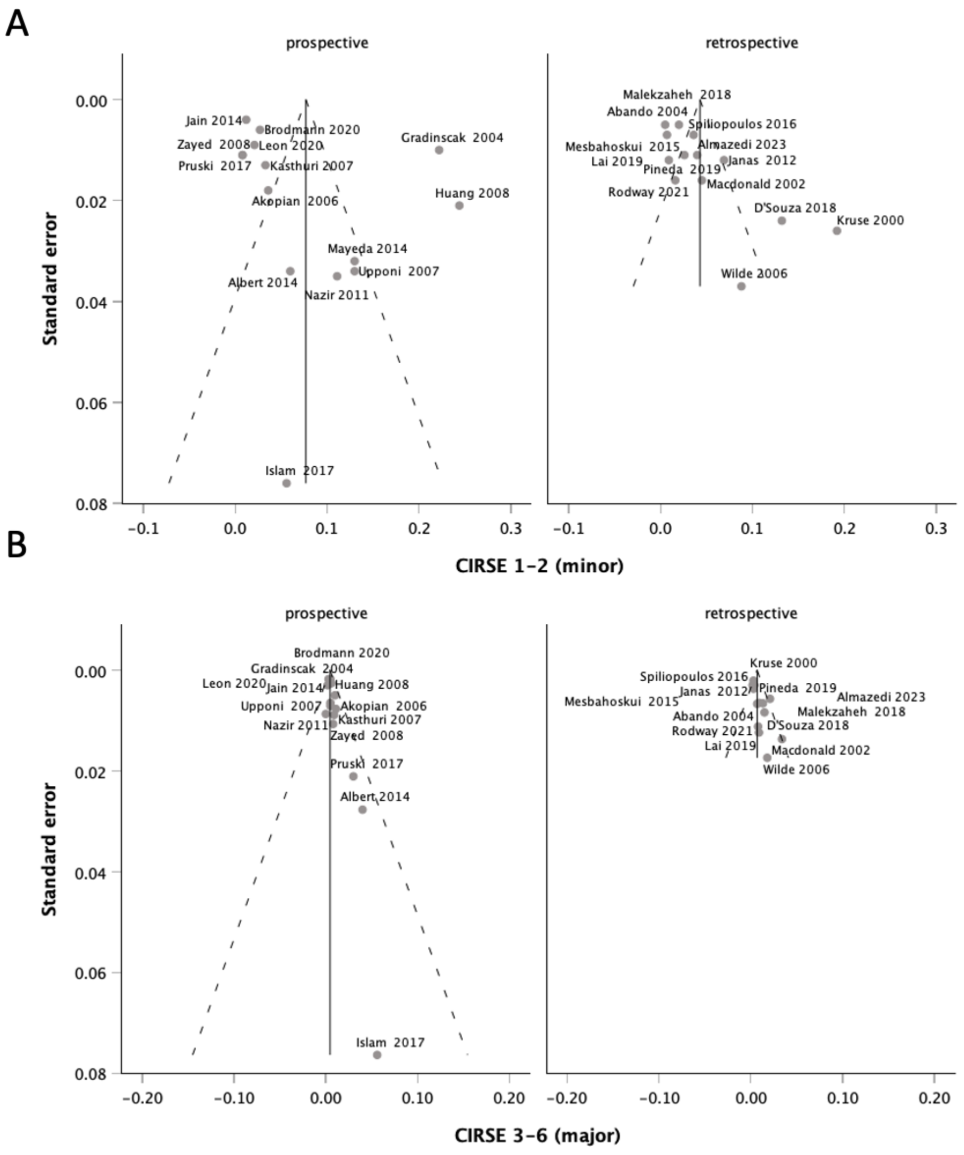

Supplementary Figure S2

References

1. Filippiadis DK, Binkert C, Pellerin O, Hoffmann RT, Krajina A, Pereira PL. Cirse Quality Assurance Document and Standards for Classification of Complications: The Cirse Classification System. *Cardiovasc Intervent Radiol* 2017; **40**(8): 1141-6.
2. Omary RA, Bettmann MA, Cardella JF, et al. Quality improvement guidelines for the reporting and archiving of interventional radiology procedures. *J Vasc Interv Radiol* 2003; **14**(9 Pt 2): S293-5.
